# NPF:network propagation for protein function prediction

**DOI:** 10.1186/s12859-020-03663-7

**Published:** 2020-08-12

**Authors:** Bihai Zhao, Zhihong Zhang, Meiping Jiang, Sai Hu, Yingchun Luo, Lei Wang

**Affiliations:** 1 College of Computer Engineering and Applied Mathematics, Changsha University, Changsha, 410022 Hunan China; 2 Hunan Provincial Key Laboratory of Industrial Internet Technology and Security, Changsha University, Changsha, 410022 Hunan China; 3 Hunan Provincial Key Laboratory of Nutrition and Quality Control of Aquatic Animals, Changsha University, Changsha, 410022 Hunan China; 4 Department of Ultrasound, Hunan Provincial Maternal and Child Health Care Hospital, Changsha, 410008 Hunan China; 5 NHC Key Laboratory of Birth Defect for Research and Prevention (Hunan Provincial Maternal and Child Health Care Hospital), Changsha, 410100 Hunan China

**Keywords:** Network propagation, Protein-protein interaction, Prediction of protein function

## Abstract

**Background:**

The accurate annotation of protein functions is of great significance in elucidating the phenomena of life, treating disease and developing new medicines. Various methods have been developed to facilitate the prediction of these functions by combining protein interaction networks (PINs) with multi-omics data. However, it is still challenging to make full use of multiple biological to improve the performance of functions annotation.

**Results:**

We presented NPF (Network Propagation for Functions prediction), an integrative protein function predicting framework assisted by network propagation and functional module detection, for discovering interacting partners with similar functions to target proteins. NPF leverages knowledge of the protein interaction network architecture and multi-omics data, such as domain annotation and protein complex information, to augment protein-protein functional similarity in a propagation manner. We have verified the great potential of NPF for accurately inferring protein functions. According to the comprehensive evaluation of NPF, it delivered a better performance than other competing methods in terms of leave-one-out cross-validation and ten-fold cross validation.

**Conclusions:**

We demonstrated that network propagation, together with multi-omics data, can both discover more partners with similar function, and is unconstricted by the “small-world” feature of protein interaction networks. We conclude that the performance of function prediction depends greatly on whether we can extract and exploit proper functional information of similarity from protein correlations.

## Background

Proteins are the main component of cells and play an essential role in nearly all cell functions such as composing cellular structure. Biological functions are performed by groups of interacting and functionally associated proteins, instead of individual proteins. The accurate characterization of protein functions is a key to understanding life at the molecular level and has a profound impact on biomedicine and pharmaceuticals.

Proteins of unknown function comprise a significant fraction of sequenced genomes [1]. Thus, how accurately unknown proteins are determined in their purposes has become one of the greatest challenges in the post-gene era. However, due to the inherent difficulty and high costs, experimental techniques to determine protein functions has been unable to meet the growing genomic sequence data. An increasing number of protein-protein interaction data urgently requires computational methods to predict protein functions.. A protein interaction network (PIN) can be modelled as an undirected graph, in which a vertex represents a protein and an edge denotes an interaction between a pair of proteins. Intuitively, numerous network-based [2–4] or graph-based [5, 6] approaches are applied to predict protein functions from PINs. These methods are based on the observation that proteins often possess similar or identical biochemical functions with their interaction partners in the PINs [2, 7]. Unfortunately, these methods are often plagued by noise and errors**,** resulting in biased outcomes and reduced confidence in PINs.

To provide an accurate prediction results, the integration of different types of biological data has become an important and popular strategy. A number of approaches have been developed to facilitate the prediction of protein functions by combining PPIs with multi-source biological data. Cozzetto et al. [8] proposed an effective method to deduce protein functions by integrating PINs with a wide variety of biological information, such as sequence, gene expression, etc. Zhang et al. [9] developed the domain context similarity for the prediction of protein functions using protein domain composition and PINs. As an improvement on Zhang’s method, two algorithms, named DCS (domain combination similarity) [10] and DSCP (domain combination similarity in context of protein complexes) were proposed to annotate unknown proteins by combining PINs with proteins’ domain information and protein complexes information. For the annotation of protein functions, the PON (protein overlap network) [11] was constructed using the protein domain information and PIN topology. Sarker et al. [12] initially reconstructed a protein-protein network based on PINs and protein domains, and then presented the *GrAPFI* method for the annotation of protein functions. INGA [13] and INGA 2.0 [14] web servers were developed to infer protein functions by combining protein interaction networks, domain assignments and sequence similarity. PANNZER2 [15] was another functional annotation web server based on sequence similarity practical. On the basis of the deep learning framework, Zhang proposed two methods: DeepGOA [16] and DeepFunc [17], for accurate prediction of protein functions. Normally, these methods are based network or neighbour-count. Multiple biological data is fused into these methods to improve the quality of the PINs. For example, Zhang, DCS and DSCP have improved neighbour-count-based methods with integrated protein domain data. The combinatorial theory was used in these three methods to calculate the functional similarity between proteins. Combining protein domain information with the topologies of PINs, the PON and *GrAPFI* method rebuilt protein interaction networks for the prediction of protein functions. Then, these two methods annotated unknown proteins according to their 1-layer neighbours in the constructed network based on neighbor count and link weights. In spite of the advances in these methods, it was a central challenge to the integration of multiple biological data categories within a single analysis framework.

**Fig. 1:**
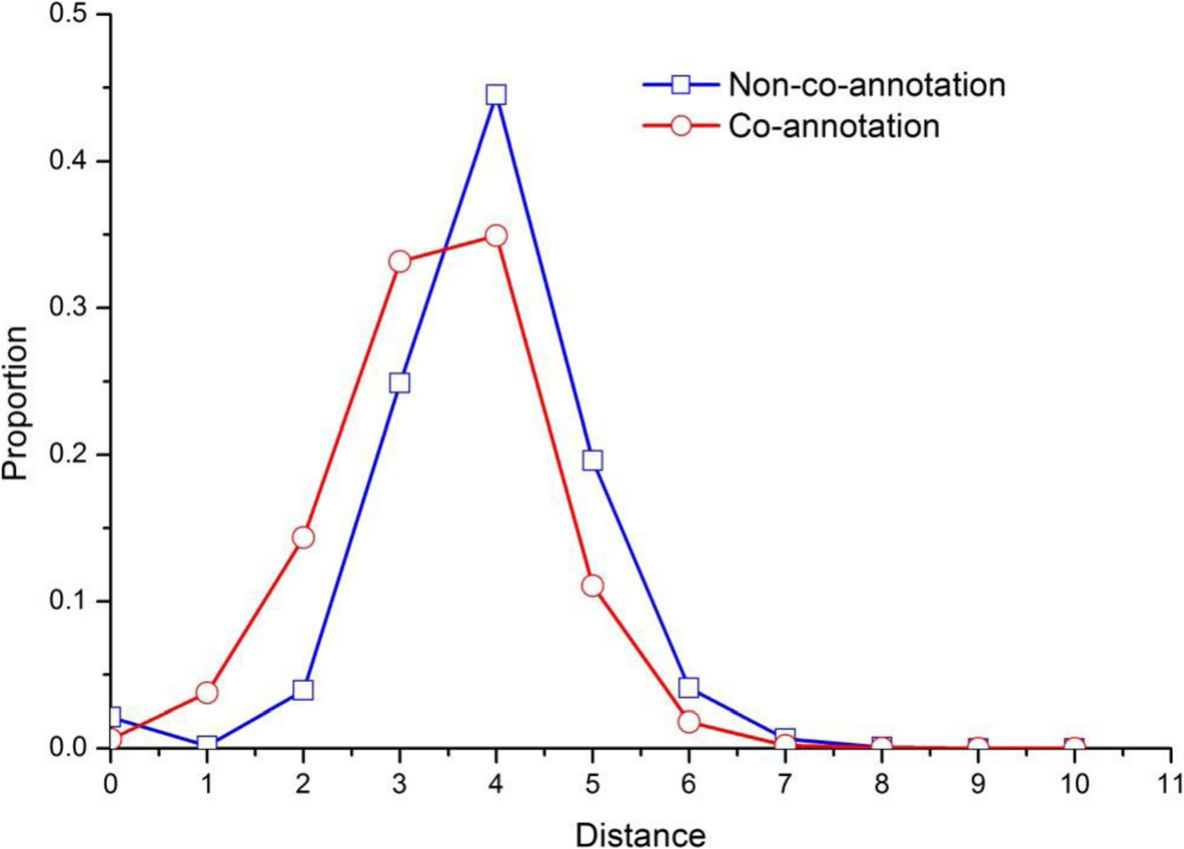
Distribution of shortest-path distances in the pairs of proteins sharing functions. This figure illustrates the shortest path length distribution of protein pairs with common functions and proteins pairs that do not share any function. The curve is plotted by short-path distances between proteins on the horizontal axis and the proportion of pairs of proteins on the vertical. The red round curve describes the relationship between pairs of proteins with common functions and the short path distances between them, and the blue rectangle curve depicts that of pairs of proteins without common functions

In the context of functions prediction, most network analysis methods depended on the principle of ‘guilt by association’, which is based on observations that a protein shares many functional features with its direct interacting partners in PINs. A simple and generic method might be to characterize unknown proteins with functions of all direct neighbours in PINs. Nevertheless, such a straightforward way would potentially yield false positives that are linked to proteins by irrelevant interactions; it would also introduce false negatives that do not directly connect to proteins with known functions [18]. It is verified by our statistics on the yeast PINs. We investigated the shortest path length distribution of protein pairs with common functions and proteins pairs that do not share any function. The statistics results were shown in Fig. 1. Figure 1 shows that the proportion of protein pairs with co-annotation is higher than that of protein pairs with none-annotation when the distance is less than 3. This indicates that co-annotated proteins are closer to each other than non-co-annotated proteins. Figure 1 also reveals an interesting phenomenon that proteins seem to co-annotate with their level-3 or level-4 neighbours instead of direct interacting partners, due to the incompleteness and fault of the PINs.

To clear this hurdle, as a proxy to a ‘functional distance’ between proteins, the short-path distance instead of Euclidean distance (i.e. the short-path distance between proteins is equal to 1) was adopted in some approaches to predict protein functions. However, most of proteins can arrive reach other proteins within a few steps because of the small-world feature of the PIN. Although these approaches can effectively suppress false negatives, it will also return many spurious functions by including irrelevant interactions. Network propagation provides us with a more refined approach by using the flow of information through network connections as a means to establish relationships between nodes [19]. There are various guises of network propagation, such as random walks on graphs [20], the Google PageRank search algorithm [21], heat diffusion processes [22], graph kernels [23], etc. In biological network, plenty of methods based on network propagation have been widely applied to essential proteins identification [24, 25], drug synergy prediction [26], tumors classification [27], disease associated genes identification [28, 29], microbe-disease associations inference [30] and protein functions prediction [31], which demonstrated that network propagation is a powerful data transformation method of broad utility in genetic research [18]. Additionally, the rationality of combining the protein domain, complex information and PINs for functions prediction is substantiated by the DCS and DSCP methods.

Inspired by these findings, we developed a network propagation-based method, named NPF, for prediction of protein functions. Our model initially simulates the random walk with restart algorithm and constructs a propagation network by integrating knowledge of the protein interaction network architecture, protein domains and protein complexes. This serves as the basis for us to detect functional modules with high coupling in the prediction of functions of unknown protein. To evaluate the performance of NPF, we apply our method and six other state-of-the-art methods for prediction of protein functions on yeast PINs. Experimental results demonstrated that NPF outperformed these competing methods, including Neighbourhood-counting (NC) [2], Zhang [9], DCS [10], DSCP [10], PON [11] and *GrAPFI* [12].

## Methods

**Fig. 2:**
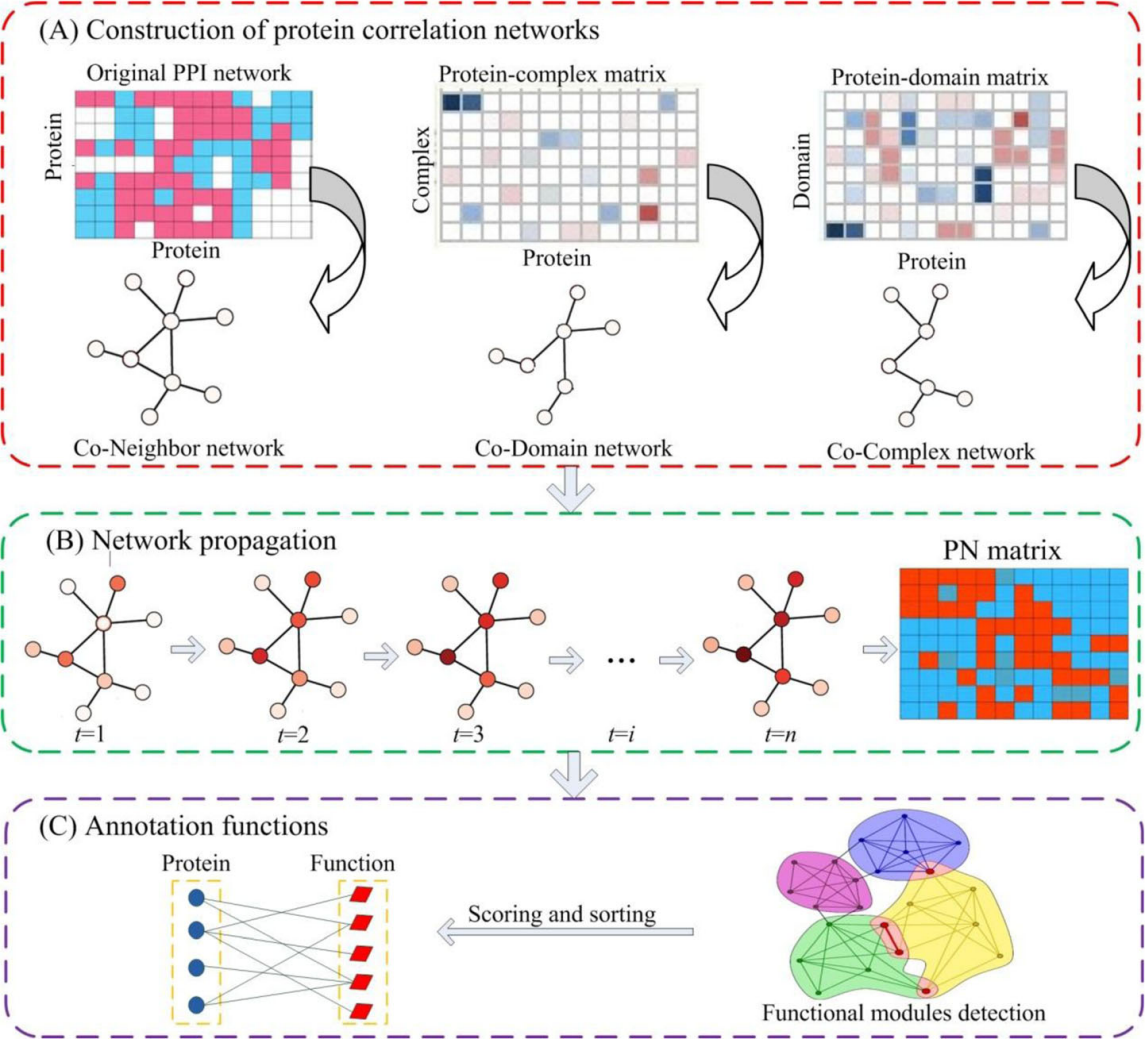
Flowchart of NPF method. **a** Three protein correlation networks Co_Neighbor, Co-Domain and Co-Complex are derived from original PIN, protein domain data, as well as protein complex information, respectively. **b** The propagation network *PN* is generated by running an improved random walk with restart algorithm on multiple functional similarity networks. The propagation process is illustrated at different steps until convergence. Changes in the color of nodes in the graph indicate the progress of the iterative process. **c** Annotation for target proteins. Taking the target node as the seed node, a highly cohesive functional module can be obtained. Functions of neighbors in the detected functional module are used to characterize the target node

The NPF method is divided into three stages: (1) Constructing three protein-protein correlation networks by integrating knowledge of the protein interaction network architecture, protein-domain associations and protein-complex associations. (2) Building a propagation network by applying an improved random walk with restart algorithm to multiple protein correlation networks. (3) Detecting functional modules with high coupling in the propagation network and annotating functions for target proteins. The flowchart for the NPF method is shown in Fig. 2.

### Construction of multiple protein correlation networks

Biological functions are performed by a group of genes or proteins which are related to one or more cellular interactions, e.g. protein-protein interaction, co-regulation, co-expression or membership of a protein complex. Physical PINs directly indicate the cooperation of proteins to drive a biological process [32]. Moreover, computational approaches had successfully detected stable functional modules from co-expression networks [33]. We suspect that tightly interacting and functionally dependent proteins may co-express, co-regulate or share a common protein complex, etc. Therefore, we constructed multiple protein-protein correlation networks with integration of knowledge of protein interaction network architecture, protein domain annotation and protein complexes information.

### Co-neighbor network

Molecular functions are performed by groups of proteins interacting to each other. Thus, a straightforward strategy is to annotate proteins for target proteins using knowledge of the protein interaction network architecture. In this study, we used the overlapping interacting partners between a pair of proteins as an estimate of their functional correlation. In the Co-Neighbor network, two proteins are connected if they have a physical interaction and link to one or more common proteins simultaneously. Given a pair of proteins *p*_*i*_ and *p*_*j*_ in the Co-Neighbor network, their correlation value was calculated as follow [34]:

1 $$ P\_N\left({p}_i,{p}_j\right)=\frac{2\mid {N}_{p_i}\cap {N}_{p_j}\mid }{\mid {N}_{p_i}\mid +\mid {N}_{p_i}\cap {N}_{p_j}\mid}\ast \frac{2\mid {N}_{p_i}\cap {N}_{p_j}\mid }{\mid {N}_{p_j}\mid +\mid {N}_{p_i}\cap {N}_{p_j}\mid } $$

where, *N*_*pi*_ and *N*_*pj*_ represents the set of direct neighbors of *p*_*i*_ and *p*_*j*_ respectively. *N*_*pi*_∩*N*_*pj*_ is an intersection of *N*_*pi*_ and *N*_*pj*_.

### Co-domain network

Domains are sequential and structural motifs found independently in different proteins and play as the stable functional block of proteins. We now generalize the idea to construct a protein correlation network based on the protein domain annotation information. For a pair of proteins *p*_*i*_ and *p*_*j*_, let *M* denotes the total number of domain categories in PINs, and let *x* and *y* represent the number of domain categories of *p*_*i*_ and *p*_*j*_, respectively. Let *z* expresses the number of overlapping domain categories between *p*_*i*_ and *p*_*j*_. Then, we measured the functional correlation between two proteins *p*_*i*_ and *p*_*j*_ in the Co-Domain network with the follow formula, which is an improvement of the Zhang method [9]:

2 $$ P\_D\left({p}_i,{p}_j\right)=-\log \frac{M^z\ast {\left(M-z\right)}^{x-z}\ast {\left(M-x\right)}^{y-z}}{M^x\ast {M}^y} $$

Finally, the correlation score between *p*_*i*_ and *p*_*j*_ was obtained by the normalization processing, which was described as follows:

3 $$ P\_D\left({p}_i,{p}_j\right)=\frac{P\_D\left({p}_i,{p}_j\right)-\min \left(P\_D\right)}{\max \left(P\_D\right)-\min \left(P\_D\right)} $$

### Co-complex network

Protein complexes consisting of molecular aggregations of proteins assembled by multiple protein interactions are fundamental units of macro-molecular organization and play crucial roles in integrating individual gene products to perform useful cellular functions. Studies [10] have revealed that if two proteins are consisted of the same protein complexes, they tend to perform the same or similar biological functions. As much, incorporating quality-controlled protein complexes and analysing functional associations are both essential for accurate function annotation. We therefore proposed to construct the protein correlation network Co-Complex, where the functional correlation between two proteins is measured using the Eq. (4) [35].

4 $$ P\_C\left({p}_i,{p}_j\right)=\frac{\mid {C}_{p_i}\cap {C}_{p_j}\mid }{\mid {C}_{p_i}\mid \ast \mid {C}_{p_j}\mid } $$

In Eq. (4), *C*_*pi*_ and *C*_*pj*_ represents the set of protein complexes in which *p*_*i*_ and *p*_*j*_ is involved respectively. *C*_*pi*_∩*C*_*pj*_ denotes the set of protein complexes containing both *p*_*i*_ and *p*_*j*_.

### Network propagation algorithm

The network propagation algorithm involved a random walk with restart process on multiple protein correlation networks to generate an aggregated protein functional similarity network with high confidence. This process considered the global connectivity patterns of the PIN for annotating target proteins. Moreover, this algorithm took into account the structural feature and modular feature of protein for measuring functional similarity by performing a two-step propagation operation. The output of the network propagation algorithm is a propagated protein functional matrix, which could be used as input for protein function prediction.

At the first step of the network propagation algorithm, we established the transition matrix, *H*, based on the Co-Neighbor network. The transition probability from protein *i* to protein *j* was computed using the following equation:

5 $$ h\left(i,j\right)=\left\{\begin{array}{ccc}\frac{P\_N\left({p}_i,{p}_j\right)}{\sum \limits_{k=1}^nP\_N\left({p}_i,{p}_k\right)}&, & if\ \sum \limits_{k=1}^nP\_N\left({p}_i,{p}_k\right)>0\\ {}0&, & otherwise\end{array}\right. $$

Intuitively, we wish to calculate functional similarity between proteins by propagation that takes both structural feature and modular feature of proteins into account. These two features are derived from domain annotation and protein complex information, respectively. Therefore, we performed a two-step propagation operation to calculate functional similarity between the protein *p*_*i*_ with other proteins by

6 $$ {VD}_i^{t+1}=\alpha \ast H\ast {VC}_i^t+\left(1-\alpha \right)\ast RV\_{D}_i $$

7 $$ {VC}_i^{t+1}=\alpha \ast {H}^T\ast {VD}_i^t+\left(1-\alpha \right)\ast RV\_{C}_i $$

where the parameter *α* ∈ [0, 1] balances between the propagation information and restart scores, *VD*^*i*^_*t*_ and *VC*^*i*^_*t*_ are two vectors at the *t* step to measure structural correlation and modular correlation between protein *p*_*i*_ with the remaining proteins, respectively. Elements of the two vectors are initialized to 1/*n* (i.e., ${VD}_i^0=\left[1/n,1/n,\cdots, 1/n\right]$^*T*^, ${VC}_i^0=\left[1/n,1/n,\cdots, 1/n\right]$^*T*^). It was to note that it is possible to tune the functional similarity scores by defining two restart vectors *RV*_*D*_*i*_ and *RV*_*C*_*i*_ by

8 $$ RV\_{D}_i={\left[P\_D\left(i,1\right),P\_D\left(i,2\right),\cdots P\_D\left(i,n\right)\right]}^T $$

9 $$ RV\_{C}_i={\left[P\_C\left(i,1\right),P\_C\left(i,2\right),\cdots P\_C\left(i,n\right)\right]}^T $$

In this study, we set α to 0.5 [36, 37]. When the propagation converges, we can obtain an adjacency matrix responding to the propagation network, which is formally described as follows:

10 $$ PN=\left[\begin{array}{cccc}{VC}_{11}+{VD}_{11}& {VC}_{12}+{VD}_{12}& \cdots & {VC}_{1n}+{VD}_{1n}\\ {}{VC}_{21}+{VD}_{21}& \ddots & & \vdots \\ {}\cdots & \cdots & \ddots & \vdots \\ {}{VC}_{n1}+{VD}_{n1}& \cdots & \cdots & {VC}_{nn}+{VD}_{nn}\end{array}\right] $$

The overall framework of network propagation algorithm can be illustrated as the Algorithm 1. The proof of convergence on the Algorithm 1 can be found in Additional file 1.

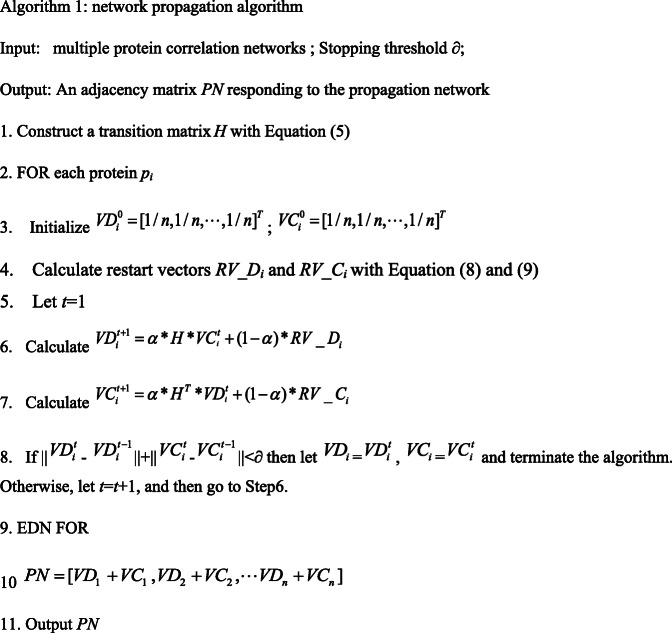



### Prediction of protein functions

Intuitively, interacting partners are helpful to characterize target proteins. However, members of the same functional module are often more densely connected than those across functional modules [38]. Therefore, at the final stage of our work, we threw out loosely connected neighbours and annotated target proteins with the remaining partners in the newly constructed propagation network. Given a target protein *v*, *M*_*V* is a module of the propagation network *PN*, which is composed of all neighbour nodes of *v*. The module fitness [39] was introduced to quantitative describe the cohesion of *M*_*V*.

11 $$ {f}_{M\_V}=\frac{WD_{M\_V}^{in}}{{\left({WD}_{M\_V}^{in}+{WD}_{M\_V}^{out}\right)}^{\beta }} $$

where *WD*^*M* _ *V*^_*in*_ denotes the total weight of edges contained entirely by a group of proteins in the module *M*_*V*, *WD*^*M* _ *V*^_*out*_ denotes the total weight of edges that connect the group with the rest of the network. β is a positive real-valued parameter, controlling the size of the module. To simplify operation, we set β to 1. The aim of this stage was to determine a module starting from protein *v* such that the inclusion of a new neighbour or the elimination of one neighbor from the module would lower *f*_*M_V*_. Thus for this purpose, we introduced the concept of neighbour fitness. Given a *v*’s neighbour *u*, the neighbour fitness of *u* in reference to the module *M*_*V* was calculated as follows:

12 $$ {f}_{M\_V}^u={f}_{M\_V+\left\{u\right\}}-{f}_{M\_V-\left\{u\right\}} $$

In eq. (12), *M*_*V*+ {*u*} and *M*_*V*-{*u*} represents the module obtained from *M*_*V* with neighbour *u* inside and outside, respectively.

First, neighbours of *v* were ranked in descending order according to the functional similarity to *v*. And then, all neighbours of *v* were visited and nodes with neighbour fitness greater than 0 were selected to form a candidate proteins set *P* = {*p*_1_, *p*_2_,…, *p*_*l*_}. Let *F* = {*f*_1_, *f*_2_,…, *f*_*m*_} be a list of functions of all proteins in *P*. The score of a candidate function *f*_*j*_ in *F* can be calculated as follows:

13 $$ Score\_F\left({f}_j\right)=\sum \limits_{u=1}^l PN\left(v,u\right)\ast {t}_{uj} $$

where *PN*(*v*, *u*) represents the functional similarity between *u* and *v* in the newly constructed propagation network. If *u* contains function *f*_*j*_, then *t*_*uj*_ = 1, otherwise *t*_*uj*_ = 0. Finally, candidate functions were ranked in descending order according to their scores and TOP *K* of them were selected to characterize the target protein *v*. In this study, the parameter *K* was set to the number of functions of the protein with the greatest functional similarity to *v* in the propagation network *PN*. The Algorithm 2 gave the overall framework of the proposed NPF method.

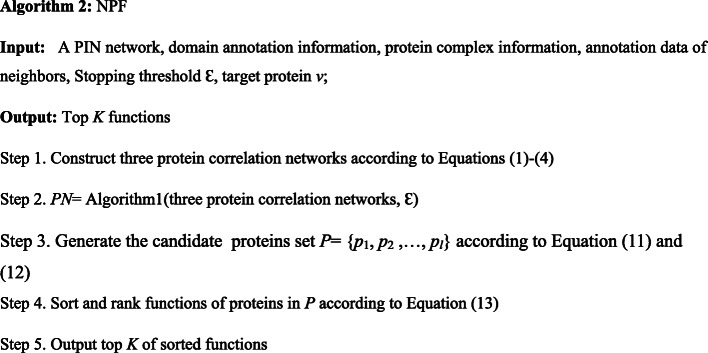



## Results

### Experimental data

To test the performance of NPF, we applied our method and six competing methods to infer protein functions in the protein interaction network of *Saccharomyces cerevisiae* (Baker’s yeast), because of their completeness, convincement, and widespread used in function prediction algorithms as gold standard data. The PIN data is derived from BioGRID database [40], updated to Oct.28, 2017, which consists of 4113 proteins and 26,105 interactions among the proteins with self-interactions and repeated interactions removed. The BioGRID is an integrated network, which has been proven successful in tasks such as predicting disease genes [41].

The annotation data of proteins used for validation was downloaded from GO official website [42]. The GO system consists of three separate categories of annotations, namely molecular function, biological process and cellular component. This paper takes the biological process as an example to analyse the performance of NGF. The protein domain data was downloaded from Pfam database [43], which contains 1026 different types of domains associated with 2566 proteins in the BioGRID network. The benchmark protein complexes set was adopted from CYC2008 [44], which consists of 408 complexes involving 1600 proteins in the BioGRID dataset. The above four dataset were uniformly transformed to use the Ensemble Genomes Protein labelling system.

### View of the constructed networks

**Fig. 3:**
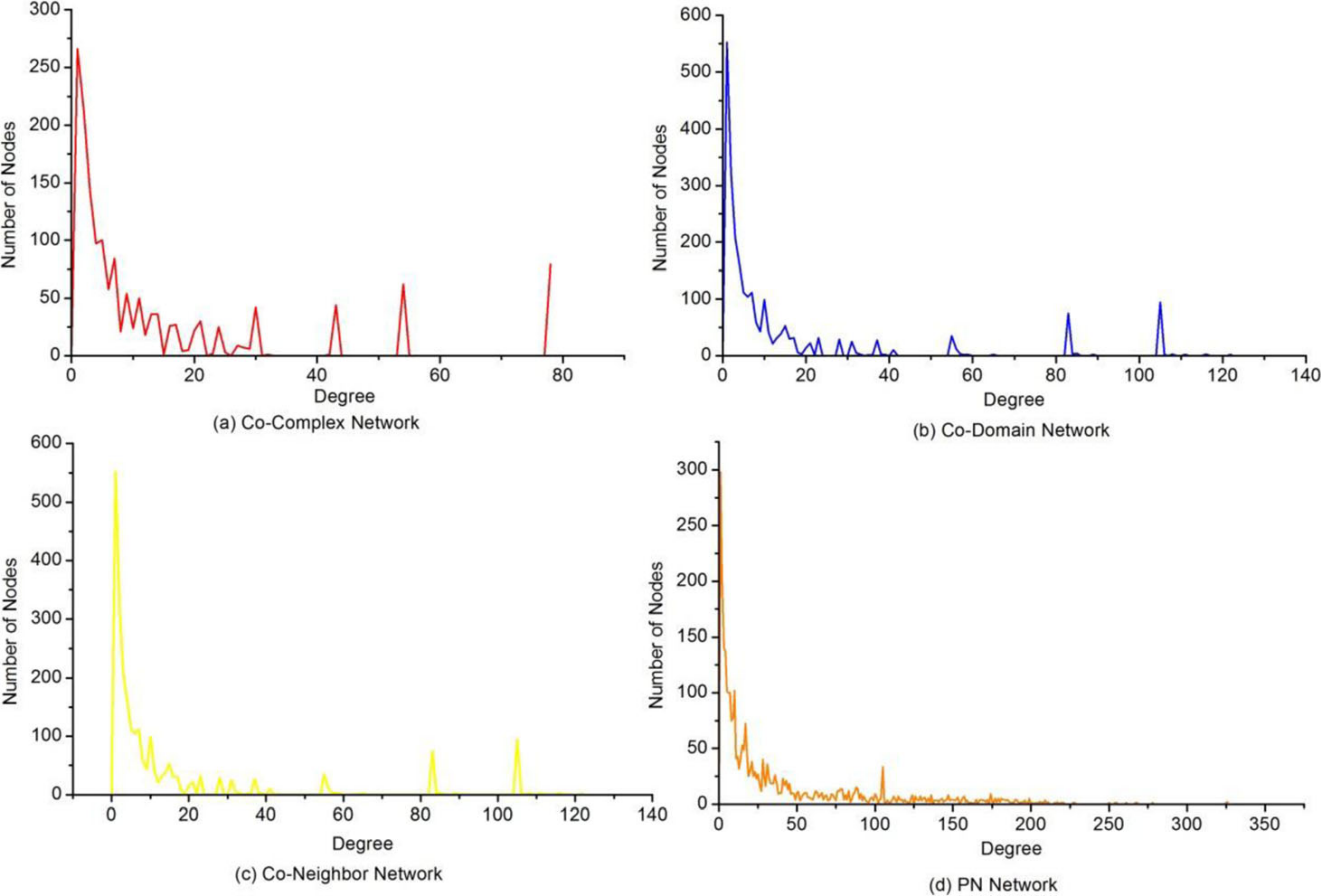
The distribution of degree in constructed networks. This Figure shows the distribution of degree in the four constructed networks. (**a**) Co-Complex network, (**b**) Co-Domain network, (**c**) Co-Neighbor network, (**d**) PN network

**Fig. 4:**
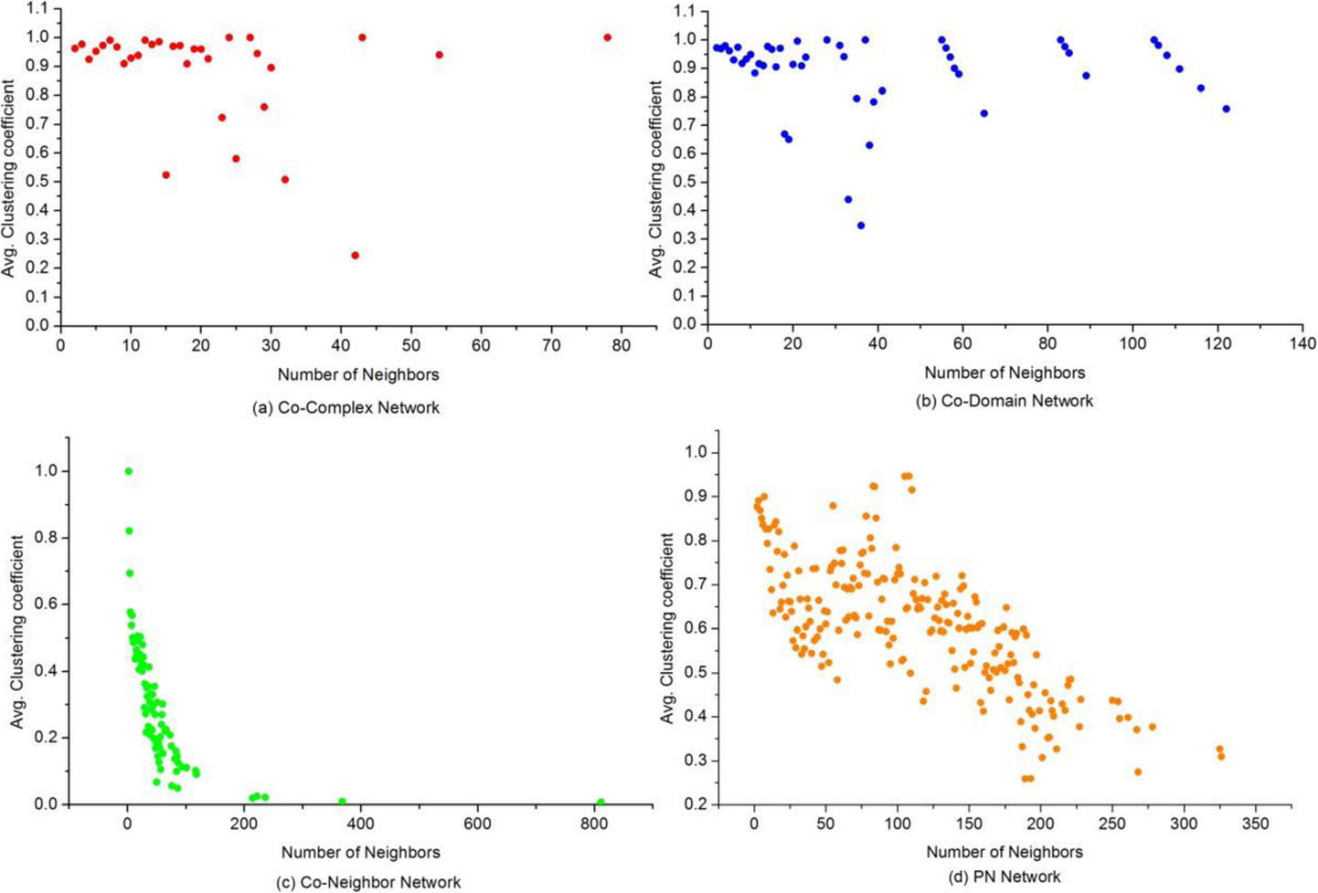
The distribution of clustering coefficient in constructed networks. This Figure shows the distribution of clustering coefficient in the four constructed networks. (**a**) Co-Complex network, (**b**) Co-Domain network, (**c**) Co-Neighbor network, (**d**) PN network

In order to better understand the behaviour of the proposed NPF method, we provided descriptive statistics on the constructed networks, including the Co-Neighbor network, Co-Domain network, Co-Complex network and PN (propagation network). Table 1 listed the basic statistics of the four constructed networks, such as size of networks, average degree etc. When considering network characteristics, the characteristic path length and the clustering coefficient are usually used to measure the network. Table 2 shows the topology features of the original PPI network and the constructed propagation network. The results indicate that the effect of the small-world characteristic on function prediction was improved through network propagation. Figures 3 and 4 depicted the distribution of degree and clustering coefficient in these four networks, respectively. Our statistics revealed reinforcing functional correlations or relationships between proteins in the PN. Therefore, it is reasonable to believe that network propagation is helpful to reduce the negative effect of false negative and improve the accuracy of prediction of protein functions.

**Table 1:** Statistics of constructed networks

Networks	Number of nodes	Number of edges	Average degree	Clustering coefficient	Connected components
Co-Neighbor	2696	13,728	10.184	0.645	25
Co-Domain	2448	18,123	14.806	0.743	471
Co-Complex	1595	10,886	13.650	0.798	279
PN	3082	57,256	37.155	0.673	185

**Table 2:** Comparison of the original network and the constructed network

Networks	Number of nodes	Number of edges	Characteristic path length	Clustering coefficient
BioGRID	4113	26,105	3.461	0.309
PN	3082	57,256	3.710	0.673

This table compares the topology features of the original network based on the BioGRID dataset and the constructed network PN

### Assessment criteria

Two assessment criteria were adopted to compare function prediction performance of the NPF with six competing methods, including NC [2], ZhangDC [9], DCS [10], DSCP [10], PON [11] and *GrAPFI* [12]. The NC method is a classic protein function annotation method, which is only based on the PIN. Zhang and DCS inferred protein functions through protein domain composition and PINs, and DSCP extends the protein functional similarity definition in DCS by combining the domain compositions of both proteins and complexes including them. PON and *GrAPFI* constructed a protein correlation network and characterized unknown proteins by integrating PINs and protein domain information.

Proteins in PINs were divided into two categories: the training set and the testing set. In one round of cross validation, the functions of each protein in the testing set are predicted according to the proteins in the training set. The validation process is repeated multiple times until each protein has a chance to become a member of the testing set. The final performances were evaluated by the average of all rounds. The first assessment criterion was leave-one-out cross-validation [10] which put one target protein into the testing set and the rest of proteins into the training set per round. However, the leave-one-out cross-validation was often plagued by many unannotated proteins in the network. Another assessment criterion used in this study was ten-fold cross validation [45], in which the proteins set was randomly divided into ten subsets, a single subset was retained for the testing set, and the remaining nine subsets were used as the training set. The cross-validation process was then repeated ten times, with each of the ten subsets used exactly once as the testing set. The ten results from the folds were then averaged to produce the final performance.

To assess the quality of predicted functions, we matched inferred functions with actual functions of target proteins. Precision and Recall were the commonly used measures to test the performance of function prediction methods. Precision is the fraction of predicted functions that are matched with known proteins while Recall is the fraction of known functions that are matched with predicted functions. In this study, true positive (TP), false positive (FP) and false negative (FN) represents the number of matched predicted functions, incorrectly matched predicted functions and missing matched known functions, respectively. Therefore, these two measures can be defined as follows:

14 $$ \Pr ecison=\frac{TP}{TP+ FP} $$

15 $$ \operatorname{Re} call=\frac{TP}{TP+ FN} $$

F-measure, as the harmonic mean of Precision and Recall, was another measure to evaluate the performance of a method synthetically, which was calculated as follows:

16 $$ F- measure=\frac{2\ast \Pr ecision\ast \operatorname{Re} call}{\Pr ecision+\operatorname{Re} call} $$

### Leave-one-out cross-validation

First, the leave-one-out cross validation was applied to verify the quality of predicted functions inferred by our NPF methods, as well as a representative set of competing methods: NC, Zhang, DCS, DSCP, PON and *GrAPFI*. To ensure impartiality, we filtered out those GO terms whose number of annotated proteins is less than 10 or more than 200 proteins. After being processed by this step, the number of GO terms is 267. Out of all the 4113 proteins in the PINs, 2716 proteins were annotated. The average and median number of GO terms for these annotated protein was 2.1 and 2, respectively. The NPF method obtained 2146 functional modules for these 2716 training proteins on the BioGRID databases. The average size and fitness value of the detected functional modules is 13.48 and 0.5625, respectively.

**Fig. 5:**
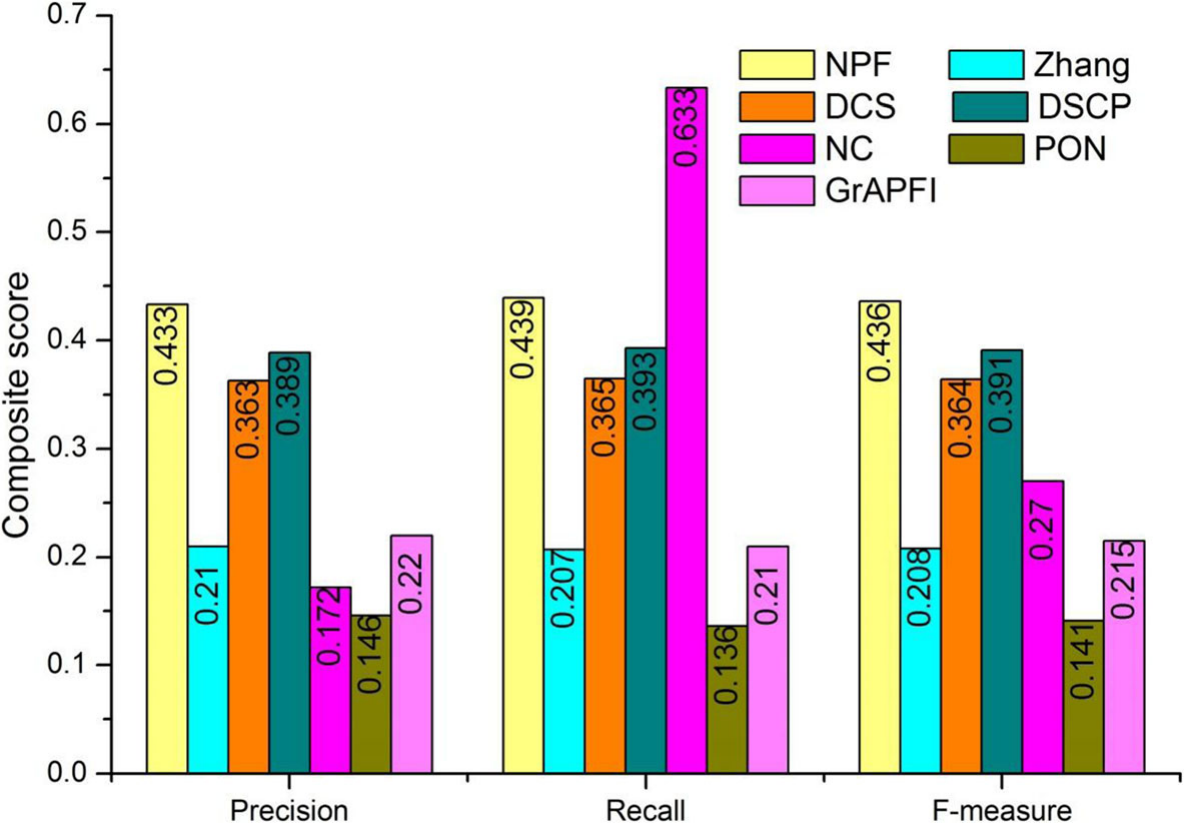
Overall comparisons of various methods. Numbers of each bar are the values for each score, including precision, recall and F-measure

We first assessed the performance of NPF and six other competing methods on these target proteins by the average Precision, Recall and F-measure. The basic information about predicted functions by NPF and six other competing methods was presented in Table 3. In Table 3, *MP* was the number of proteins successfully matching at least one known function, while *PMP* represented the number of proteins perfectly matching the known functions, yet *ZP* was the number of proteins with zero-error prediction. MMP denoted the number of proteins completely mismatching the known functions. In other words, none of the predicted functions match the known functions. From Table 3, we can see that NPF contained the second-biggest number of perfect matching proteins (891) after NC (1428), while *ZP* of our method (885) is far more than NC’s (100). Figure 5 showed the overall comparison in terms of Precision, Recall and F-measure. It illustrated that NPF archives the largest value of Precision and F-measure, the second-largest value of Recall after NC. This is due to the maximum number of perfect matching proteins with NC. F-measure of NPF was 61.56, 109.41, 19.74, 11.53, 209.80 and 103.36% higher than NC, Zhang, DCS, DSCP, PON and *GrAPFI*, respectively.

**Table 3:** Basic information of prediction by various algorithms

Methods	MP	PMP	MMP	ZP
NPF	1503	891	1213	885
NC	1945	1428	771	100
Zhang	727	421	1989	432
DCS	1269	743	1447	742
DSCP	1358	810	1358	799
PON	536	229	2180	277
*GrAPFI*	774	384	1942	432

This table shows the basic information of the results predicted by NPF, NC, Zhang, DCS, DSCP, PON and *GrAPFI*. MP is the number of proteins successfully matching at least one known function. PMP represents the number of proteins perfectly matching the known functions. MMP denotes the number of proteins completely mismatching the known functions. ZP is the number of proteins with zero-error prediction. That is, all the predicted functions in these proteins match the known functions

**Fig. 6:**
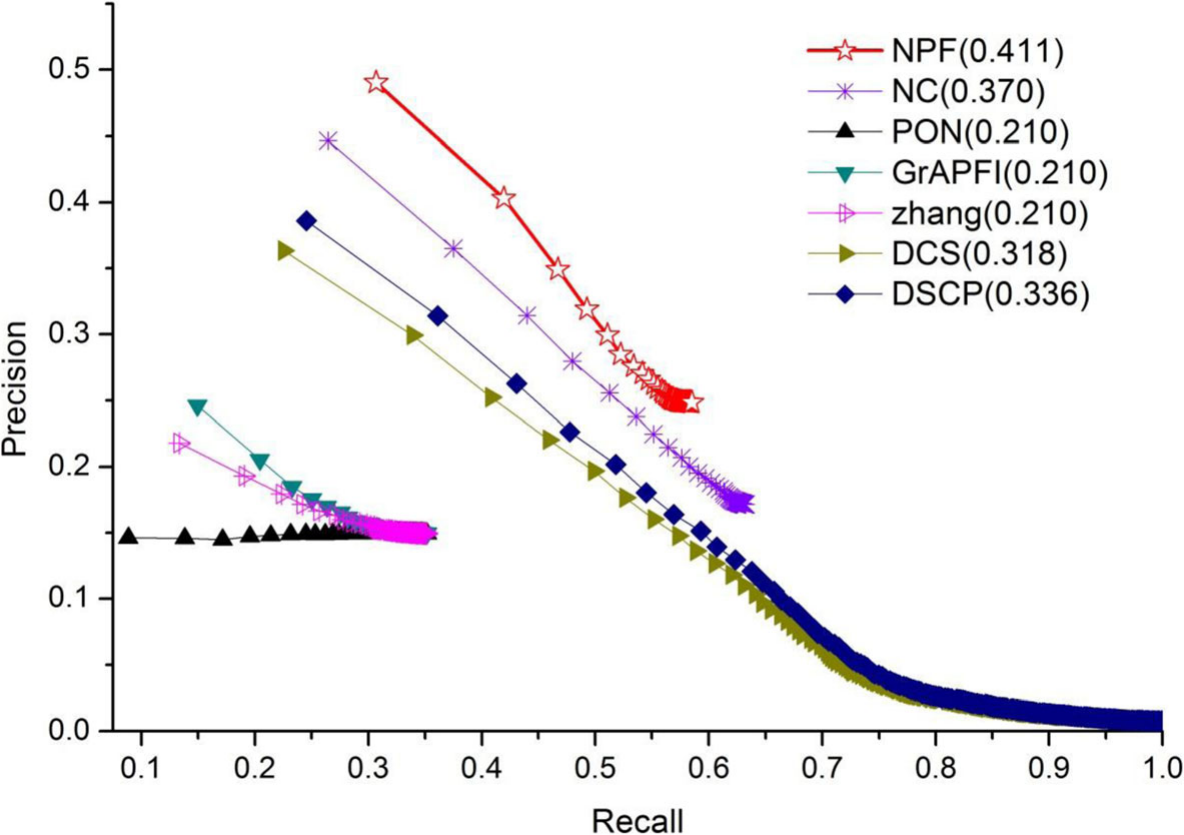
The precision-recall curves of NPF compared to six other competing methods. The figure denotes the precision-recall (PR) curves of NPF and six other competing methods (Zhang, DCS, DSCP, PON and *GrAPFI*) based on the average prediction performance over all testing protein. The vertical and horizontal coordination of the PR curves are the values of Precision and Recall, respectively. Numbers in brackets represent the maximum F-measures for these seven methods

To further investigate the performance of NPF and six other competing methods, we applied the Precision-Recall (PR) curve, whose vertical and horizontal coordination are the values of Precision and Recall, respectively. The PR curve is a standard for evaluation of the comprehensive performance of all methods in terms of different strategies of function selection. Predicted functions were ranked in descending order according to the values of functional similarity calculated by NPF, NC, PON and *GrAPFI*, respectively. Then, the top *K* functions were selected and annotated target proteins. The Parameter *K* changed from 1 to 267. As for the methods of Zhang, DSC and DSCP, top *N* (*N* < =*K*) proteins which had the highest similarity value with target proteins were selected and *K* functions in these fell out proteins were selected in turn to characterize target proteins. For a given target protein and the parameter *K*, the precision and recall values can be calculated according to the definition in Eqs. (14) and (15). The final PR curves of NPF and six other competing methods were drew according to the average precision and recall values over all target proteins. The PR curves of seven methods were illustrated in Fig. 4. Numbers in brackets represented the maximum F-measures for these seven methods. As shown in Fig. 6, NPF archived the first maximum F-measures in all methods. The PR curves of our method was above that of six other competing methods, which means that the NPF has a higher number of true positives and at the same time a smaller number of false positives when selecting different parameters. With the constant increase of *K*, the PR curve of NPF did not show drastic fluctuations. Even in the worst case, the precision value of NPF can still archive 0.248. However, the precision values of DSCP and DCS dropped sharply with the emergence of a large number of similar proteins.

**Fig. 7:**
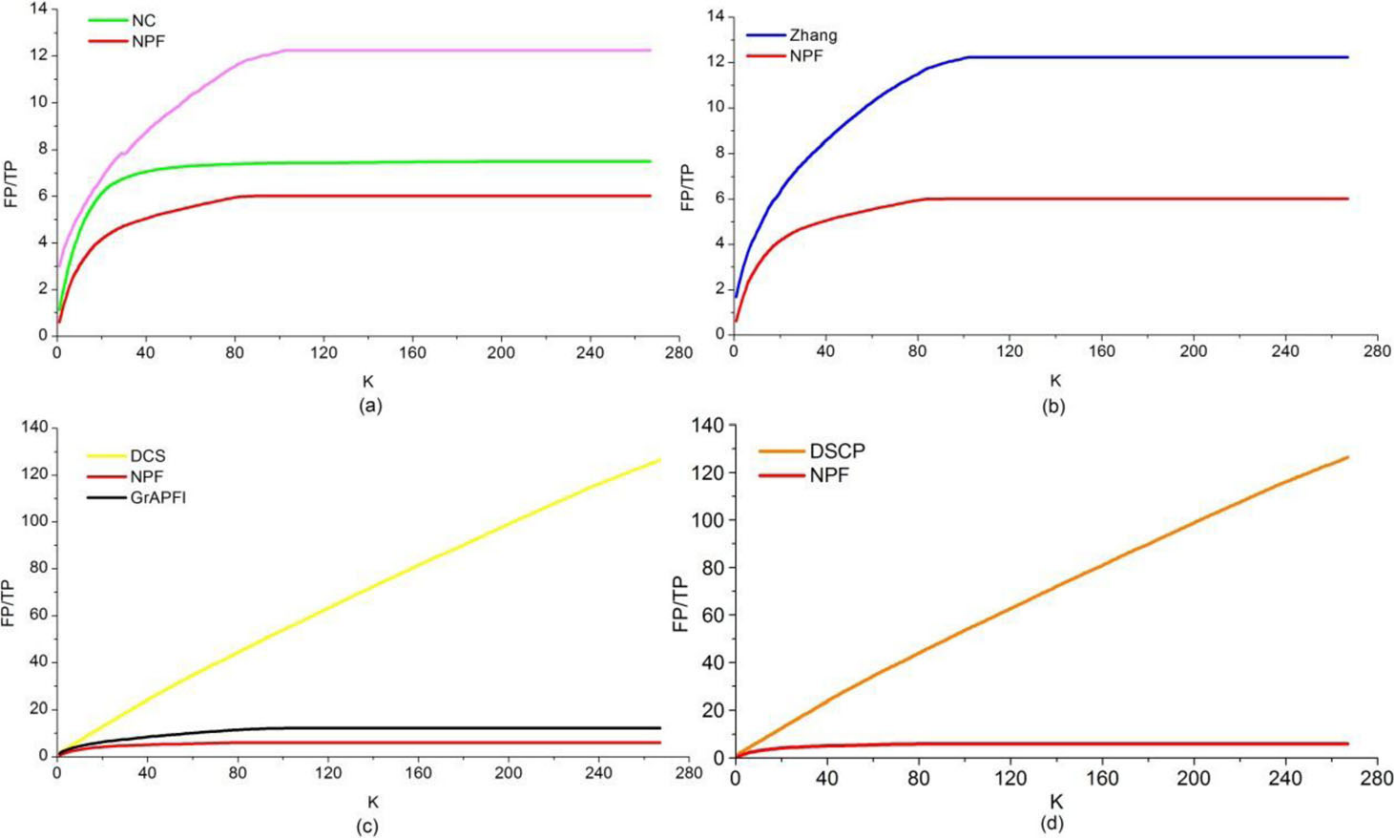
The FP/TP curves of various methods. This Figure depicts the FP/TP of our method and other competing methods fluctuate under various value of the parameter K. The vertical and horizontal coordination of the curve are the values of FP/TP and K, respectively. **a** Shows the FP/TP curves of NC and NPF. **b** Shows the FP/TP curves of Zhang and NPF. **c** Shows the FP/TP curves of DCS, *GrAPFI* and NPF. **d** Shows the FP/TP curves of DSCP and NPF

For overall comparison, we counted the number of true positive and false positive functions predicted by NPF and competing methods. A more valuable comparison between these methods was presented by plotting FP/TP curves as parameter *K* varies. Fig. 7 showed the FP/TP of our method and six other competing methods fluctuated under various value of the parameter *K* (ranging from 1 to 267). The smaller slope of the FP/TP curve of a method was, the lower the noise ratio was, which resulted in a greater predicted accuracy of the method. From this figure we can see that, FP/TP curve of NPF has consistently been covered with that of all other methods. That is, NPF generated the fewest false positives among all the methods when matching the same number of known functions.

**Fig. 8:**
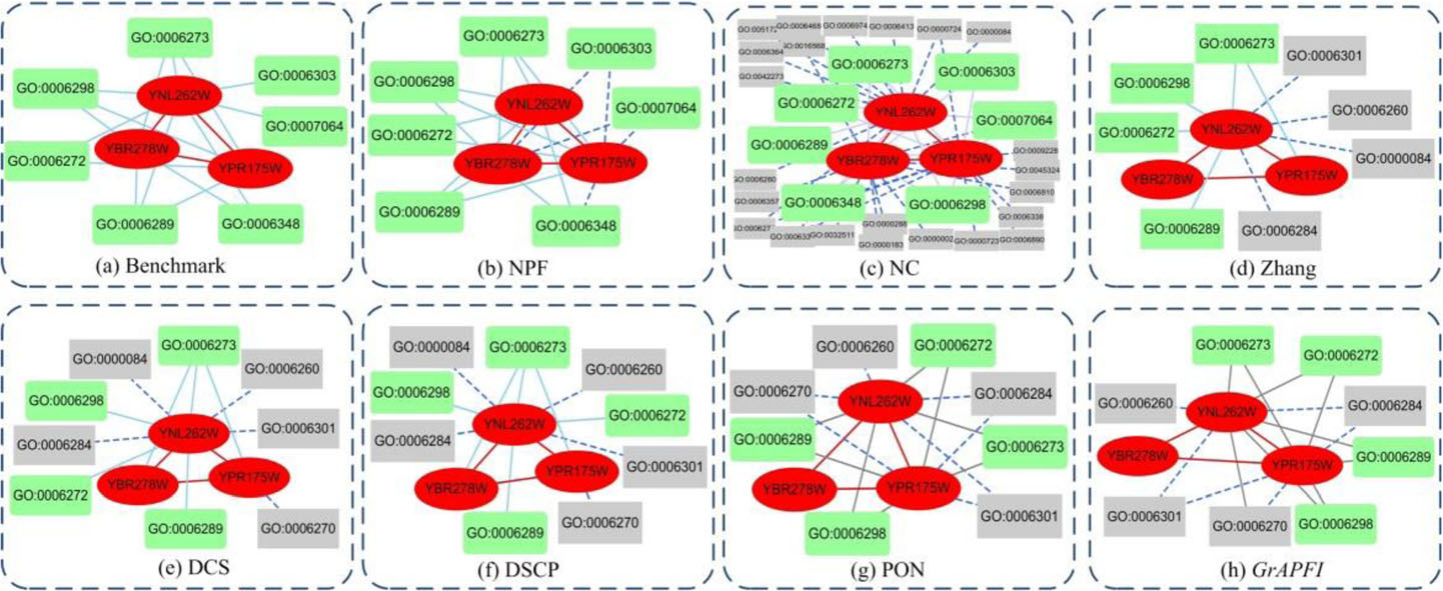
Functions of three selected proteins predicted by various methods. Red elliptic nodes denote target proteins, and red edges represent interactions between them. Green round rectangle and gray rectangle nodes represent matched functions and false matched functions respectively. Solid edges and dash edges between proteins and functions denote correct and false associations. (**a**) Benchmark results (**b**)-(**h**) is the result generated by NPF, Zhang, DCS, DSCP, PON and *GrAPFI*, respectively

To further analyze the difference between NPF and six other competing methods, we selected YNL262W, YBR278W and YPR175W as examples and inferred proteins using the seven methods. Table 4 listed the basic information of these target proteins, including degree, number of domains and number of involving complexes. Figure 8 showed the predicted functions by various methods and the benchmark set. In Fig. 8, red elliptic nodes were target proteins, and red edges represented interactions between target proteins. Green round rectangle and grey rectangle nodes represented matched functions and false matched functions, respectively. Solid edges and dash edges between proteins and functions denoted correct and false associations. Table 5 showed the description of seven known functions of the three selected proteins. Take the protein YBR278W as an example, which does not contain any domains. For the three domain-based methods Zhang, PON and *GrAPFI*, no one function was inferred, let alone matched a known function. DCS and DSCP generated two predicted functions with one function matched by including neighbors or complex members for calculation of domain context similarities. The NC method annotated the protein YBR278W with functions of its all neighbors. Although the method successfully matched five functions, it introduced a large number of false-positive functions. Out of seven functions predicted by NPF, five functions were matched with known functions. This is due to the fact that we discovered more partners with similar functions through network propagation and got rid of some functionally unrelated proteins by detecting functional modules with high coupling. The example exhibited the highest predicting accuracy of NPF, compared to the results archived by other competing methods.

**Table 4:** Basic information of selected target proteins

Proteins	Degree	Number of domains	Number of complexes
YNL262W	13	2	1
YBR278W	9	0	1
YPR175W	9	1	1

This table shows the basic information of three target proteins. The second column represents the number of its direct neighbors in the original PINs, while the third column is the number of domains it contains. The last column denotes the number of complexes involved

**Table 5:** Description of selected GO Terms

GO Term	Description
GO:0006272	Leading strand elongation, which is continuous as it proceeds in the same direction as the replication fork.
GO:0006273	Lagging strand elongation proceeds by discontinuous synthesis of short stretches of DNA, known as Okazaki fragments, from RNA primers; these fragments are then joined by DNA ligase.
GO:0006289	Nucleotide excision repair recognizes a wide range of substrates, including damage caused by UV irradiation and chemicals.
GO:0006298	The mismatch repair system promotes genomic fidelity by repairing base-base mismatches, insertion-deletion loops and heterologies generated during DNA replication and recombination.
GO:0006303	The repair of a double-strand break in DNA in which the two broken ends are re-joined with little or no sequence complementarity.
GO:0006348	Chromatin silencing at telomere means the repression of transcription of telomere DNA by altering the structure of chromatin.
GO:0007064	Mitotic sister chromatid cohesion. The cell cycle process in which the sister chromatids of a replicated chromosome are joined along the entire length of the chromosome.

The underscored text represents the name of GO Term

### Ten-fold cross validation

**Fig. 9:**
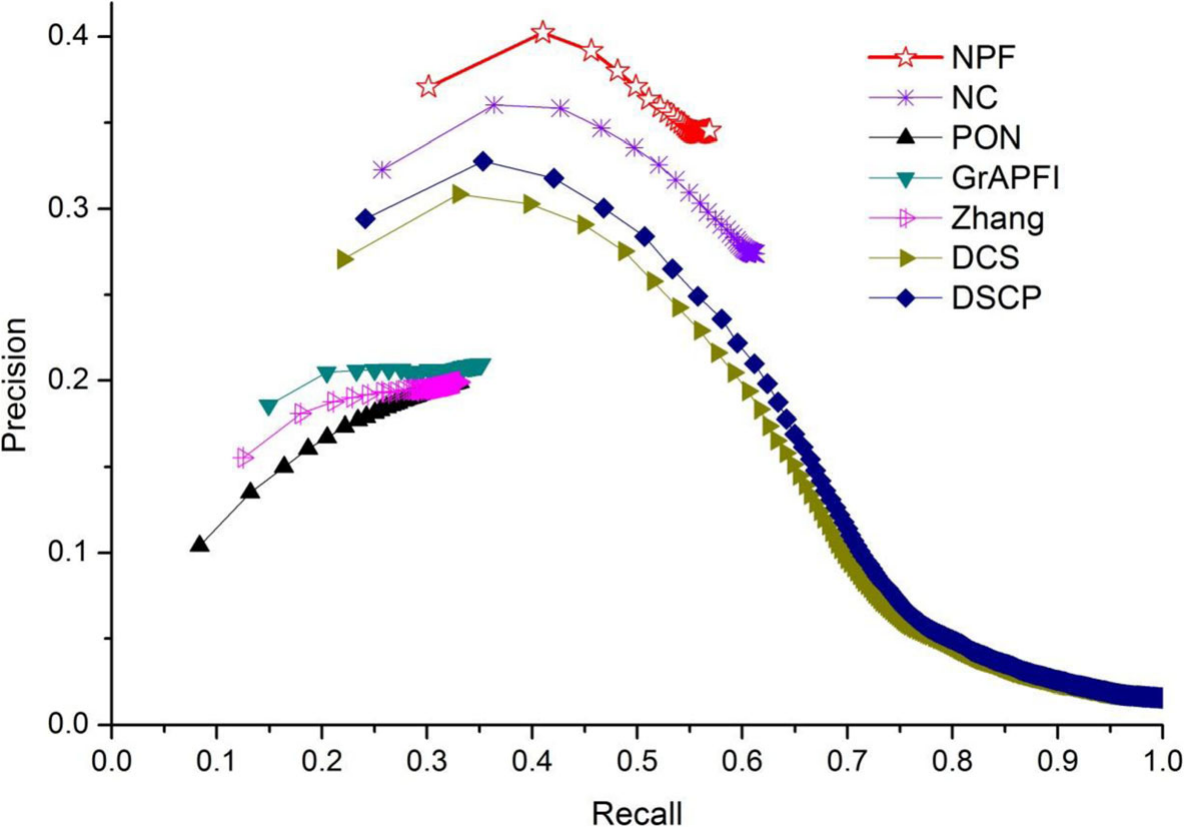
The precision-recall curves of various methods using ten-fold validation. This Figure shows the PR curves of NPF and six other methods using ten-fold validation. The entire set of proteins is divided into ten equal sets randomly, nine of which are used for training and the remaining part is used for testing. The process is repeated 1000 times, each time using another testing set

**Fig. 10:**
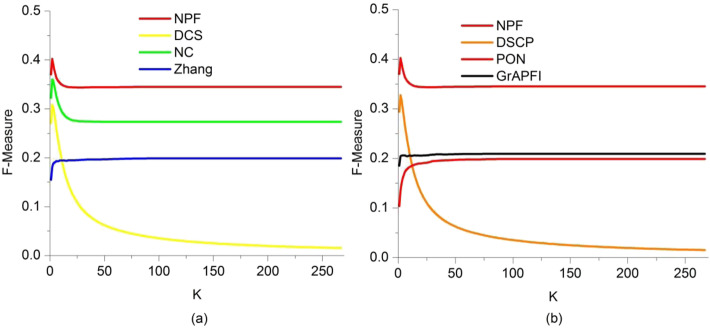
The F-measure curves of various methods using ten-fold validation. This Figure depicts the F-measure of seven methods fluctuate under various value of the parameter K. The vertical and horizontal coordination of the curve are the values of F-measure and K, respectively. **a** Shows the F-measure curves of NC, DCS, Zhang and NPF. **b** Shows the F-measure curves of DSCP, *GrAPFI*, PON and NPF

In the previous section, we applied the leave-one-out cross-validation to exhibit the NPF’s improvement on function prediction compared to the state-of-the-art methods. However, in real-world applications, there are usually much more unknown proteins than just one. To do this we adopted the ten-fold validation to verify the validity of our method on PINs with less function information. The entire set of proteins was divided into ten equal sets randomly, nine of which were used for training and the remaining part was used for testing. The process is repeated 1000 times, each time using another testing set. We ran the functional annotation methods of NPF, Zhang, DCS, DSCP, PON and *GrAPFI* on PINs to get mean values and standard deviations of precision, recall and F-measure, as shown in Table 6. Additionally, predicted functions were ranked in descending order according to the values obtained by various method and the top K functions were selected to annotate target proteins. A more valuable comparison between these methods was presented by plotting PR curves and F-measure curves as the parameter K varies using the ten-fold validation. Figures 9 and 10 illustrated the PR curves and F-measure curves of various methods, respectively. Table 6, Figs. 9 and 10 exhibited the performance improvement of NPF compared to six other competing methods. Therefore, NPF seemed to be an effective method for characterizing unknown proteins.

**Table 6:** The prediction results using ten-fold validation

Methods	Precison		Recall		F-measure	
	mean value	standard deviations	mean value	standard deviations	mean value	standard deviations
NPF	0.424	0.025	0.429	0.022	0.426	0.022
NC	0.176	0.014	0.610	0.023	0.273	0.018
Zhang	0.198	0.019	0.196	0.019	0.197	0.019
DCS	0.352	0.025	0.354	0.027	0.353	0.025
DSCP	0.378	0.027	0.382	0.028	0.380	0.027
PON	0.139	0.017	0.129	0.016	0.134	0.016
*GrAPFI*	0.219	0.018	0.209	0.018	0.214	0.018

## Discussions

The accurate annotation of protein functions is the key to understanding life at the molecular level and plays an important role in disease treatment, new drug development. Limited by the quality of protein interaction data generated by high-throughput technologies, methods that infer protein functions in terms of protein interactions may not work well [10]. A popular optimization scheme for the problem is to infer protein functions by combining PINs with multiple biological data. Despite the advances in these methods, designing efficient algorithms to fuse these multi-source biological data remains challenging. Additionally, the topology of the PINs, such as the “small world”, is also one of the factors that affect the prediction performances. Here, we presented the NPF, a network propagation-based method to annotate functions for target proteins. To overcome the problem of incomplete and false interaction data, we constructed a propagation network by integrating knowledge of the protein interaction network architecture, protein-domain associations and protein-complex associations. By propagating functional similarities across the networks, we can obtain more functionally relevant interacting partners to characterize the target proteins, which effectively free from the constraints of the “small-world” characteristic. Additionally, we take out those redundant function-independent partners by forming functional modules with high cohesion. Comprehensive comparisons among the state-of-the-art methods and our method have been made in terms of the leave-one-out cross-validation and the ten-fold cross validation. Experimental results demonstrated that our method outperforms other competing methods. Specially, DSCP used the same kind of data as NPF, yet NPF outperformed DSCP. There are two reasons to believe that NPF probably come out much better in the comparison. On the one hand, NPF can discover more neighbors with similar functions through network propagation; on the other hand, NPF predicted functions using multiple neighbors, not just the closest neighbors. In my opinion, proteins may be involved in different functional modules to perform multiple biological functions. Based on these results, we can conclude that the network propagation is useful for the study of protein interaction networks.

## Conclusions

In this study, we proposed a novel protein functions annotation method based on network propagation, named NPF, which incorporates the topology of PINS and multiple biological data, such as domain annotation information, protein complexes information. Furthermore, we guarantee the NPF against false functions by detecting functional modules based on the neighbour fitness. Experimental comparison results between NPF and six state-of-the-art methods on yeast PINs showed that NPF significantly outperforms other competing methods. In our future study, we will take the hierarchical structure of GO Terms into account for further improvement of the performance of function prediction.

## Supplementary information

**Additional file 1 Algorithm convergence**. This file provides the proof of the Algorithm 1 convergence about the effect of parameter α and *∂* on the speed of convergence.

## Data Availability

Publicly available datasets were analysed in this study. This data and the NGF program can be found here: https://github.com/husaiccsu/NPF.

## Abbreviations

PIN: Protein interaction network
NPF: Network propagation for functions prediction
DCS: Domain combination similarity
DSCP: Domain combination similarity in context of protein complexes
PON: Protein overlap network
INGA: Interaction network go annotator
PANNZER: Protein ANNotation with Z-scoRE
NC: Neighbourhood-counting
PN: Propagation network
GO: Gene ontology
TP: True positive
FP: False positive
FN: False negative
