# Supplementary material for: NPF:network propagation for protein function prediction
Source: BMC Bioinformatics. 2020 Aug 12;21:355. doi: 10.1186/s12859-020-03663-7 (PMC7430911; doi:10.1186/s12859-020-03663-7)
Supplement: Supplementary file 1 — Additional file 1 Algorithm convergence. This file provides the proof of the Algorithm 1 convergence about the effect of parameter α and ∂ on the speed of convergence. [file 12859_2020_3663_MOESM1_ESM.pdf]

Algorithm convergence

THEOREM 1. Given matrix H and vectors RV_D_i, RV_C_i, VC_i^0 and VD_i^0, the algorithm converges. The number of iteration depends on parameters α and ∂.

PROOF. The convergence of the algorithm depends on Equation (6) and (7). Equation (6) converges if and only if $\rho(\alpha H)<1$ and Equation (7) converges if and only if $\rho(\alpha H^T)= \rho(\alpha H)<1$ ($H=H^T$). $\rho(\alpha H)$ is the spectral radius of matrix αH. Since $h(i,j)\geq 0$, and for each row i of the matrix H, either $\sum_{j=1}^{n} h(i,j)=1$ or $\sum_{j=1}^{n} h(i,j)=0$, $\rho(H)=\|H\|_1$. It is easy to see that $\rho(\alpha H)= \alpha\rho(H)<1$ ($0<\alpha<1$).

Therefore, Equation (6) and (7) converge and so does the Algorithm1.

Furthermore, according to Equation (6) and (7),

$$VD_i^t = \alpha HVC_i^{t-1} + (1-\alpha)RV\_D_i \qquad VC_i^t = \alpha H^T VD_i^{t-1} + (1-\alpha)RV\_C_i$$

$$VD_i^{t-1} = \alpha HVC_i^{t-2} + (1-\alpha)RV\_D_i \qquad VC_i^{t-1} = \alpha H^T VD_i^{t-2} + (1-\alpha)RV\_C_i$$

And thus, we can get:

$$\begin{aligned} VD_i^t - VD_i^{t-1} &= \alpha H(VC_i^{t-1} - VC_i^{t-2}) \\ &= \cdots \\ &= \begin{cases} (\alpha H)^{t-1}[\alpha HVC_i^0 - (1-\alpha)RV\_D_i - VD_i^0] & , \ if\ t \bmod 2=1 \\ (\alpha H)^{t-1}[\alpha HVD_i^0 - (1-\alpha)RV\_C_i - VC_i^0] & , \ otherwise \end{cases} \end{aligned}$$

$$\begin{aligned} VC_i^t - VC_i^{t-1} &= \alpha H^T(VD_i^{t-1} - VD_i^{t-2}) = \alpha H(VD_i^{t-1} - VD_i^{t-2}) \\ &= \cdots \\ &= \begin{cases} (\alpha H)^{t-1}[\alpha HVD_i^0 - (1-\alpha)RV\_C_i - VC_i^0] & , \ if\ t \bmod 2=1 \\ (\alpha H)^{t-1}[\alpha HVC_i^0 - (1-\alpha)RV\_D_i - VD_i^0] & , \ otherwise \end{cases} \end{aligned}$$

Therefore,

$$|VD_i^t - VD_i^{t-1}| + |VC_i^t - VC_i^{t-1}|$$

$$=|(\alpha H)^{t-1}[\alpha HVC_i^0 - (1-\alpha)RV\_D_i - VD_i^0]| + |(\alpha H)^{t-1}[\alpha HVD_i^0 - (1-\alpha)RV\_C_i - VC_i^0]|$$

$$= (\alpha H)^{t-1}(|[\alpha HVC_i^0 - (1-\alpha)RV\_D_i - VD_i^0]| + |[\alpha HVD_i^0 - (1-\alpha)RV\_C_i - VC_i^0]|)$$

$$\leq \alpha^{t-1}(|[\alpha HVC_i^0 - (1-\alpha)RV\_D_i - VD_i^0]| + |[\alpha HVD_i^0 - (1-\alpha)RV\_C_i - VC_i^0]|) \leq \partial,$$

and

$$t \geq \log(\partial / (|[\alpha HVC_i^0 - (1-\alpha)RV\_D_i - VD_i^0]| + |[\alpha HVD_i^0 - (1-\alpha)RV\_C_i - VC_i^0]|)) / \log \alpha$$

Clearly, given matrix H and vectors RV_D_i, RV_C_i, VC_i^0 and VD_i^0, the number of iteration depends on parameters α and ∂.
